# The Birth of the Mammalian Sleep

**DOI:** 10.3390/biology11050734

**Published:** 2022-05-11

**Authors:** Rubén V. Rial, Francesca Canellas, Mourad Akaârir, José A. Rubiño, Pere Barceló, Aida Martín, Antoni Gamundí, M. Cristina Nicolau

**Affiliations:** 1 Laboratori de Neurofisiologia del Son i dels Ritmes Biològics, Grup de Recerca Neurofisiologia del Son i Ritmes Biològics, Department of Biologia, Universitat de les Illes Balears, Ctra Valldemossa, km 7.5, 07122 Palma de Mallorca, Illes Balears, Spain; francesca.canellas@ssib.es (F.C.); mourad.akaarir@uib.es (M.A.); josepsico78@hotmail.com (J.A.R.); pbarcelocaldentey@gmail.com (P.B.); aida.martin.reina@gmail.com (A.M.); antoni.gamundi@uib.es (A.G.); cristina.nicolau@uib.es (M.C.N.); 2 IdISBa, Institut d’Investigació Sanitària de les Illes Balears, Hospital Son Espases, 07120 Palma de Mallorca, Illes Balears, Spain; 3 IUNICS, Institut Universitari d’Investigació en Ciències de la Salut, Hospital Universitary Son Espases, 07120 Palma de Mallorca, Illes Balears, Spain

**Keywords:** evolutionary bottleneck, evolution of sleep, sleep variability, wakeful idling, function of sleep

## Abstract

**Simple Summary:**

Mammals evolved from reptiles as a consequence of an evolutionary bottleneck. Some diurnal reptiles extended their activity, first to twilight and then to the entire dark time. This forced the change of the visual system. Pursuing maximal sensitivity, they abandoned the filters protecting the eyes against the dangerous diurnal light, which, in turn, forced immobility in lightproof burrows during light time. This was the birth of the mammalian sleep. Then, the Cretacic-Paleogene extinction of dinosaurs leaved free the diurnal niche and allowed the expansion of a few early mammals to diurnal life and the high variability of sleep traits. On the other hand, we propose that the idling rest is a state showing homeostatic regulation. Therefore, the difference between behavioral rest and wakeful idling is rather low: both show quiescence, raised sensory thresholds, reversibility, specific sleeping-resting sites and body positions, it is a pleasing state, and both are dependent of circadian and homeostatic regulation. Indeed, the most important difference is the unconsciousness of sleep and the consciousness of wakeful idling. Thus, we propose that sleep is a mere upgrade of the wakeful rest, and both may have the same function: guaranteeing rest during a part of the daily cycle.

**Abstract:**

Mammals evolved from small-sized reptiles that developed endothermic metabolism. This allowed filling the nocturnal niche. They traded-off visual acuity for sensitivity but became defenseless against the dangerous daylight. To avoid such danger, they rested with closed eyes in lightproof burrows during light-time. This was the birth of the mammalian sleep, the main finding of this report. Improved audition and olfaction counterweighed the visual impairments and facilitated the cortical development. This process is called “The Nocturnal Evolutionary Bottleneck”. Pre-mammals were nocturnal until the Cretacic-Paleogene extinction of dinosaurs. Some early mammals returned to diurnal activity, and this allowed the high variability in sleeping patterns observed today. The traits of Waking Idleness are almost identical to those of behavioral sleep, including homeostatic regulation. This is another important finding of this report. In summary, behavioral sleep seems to be an upgrade of Waking Idleness Indeed, the trait that never fails to show is quiescence. We conclude that the main function of sleep consists in guaranteeing it during a part of the daily cycle.

## 1. Introduction

Many sleep-related questions remain unknown. It is generally recognized, however, that an important part of our ignorance would be resolved if we would know, with precise details, how sleep evolved. Wanting to know the present state of this question, we analyzed several recent reports (since 2016) trying to explain the evolution of sleep. [1,2,3,4,5,6,7,8,9,10,11]. With no exception, all authors consider that sleep is monophyletic, i.e., it seems that appeared in a very primitive animal and, since then, their descendants inherited the need to sleep. All authors also believe that sleep provides some vital advantage: animals that did sleep, survived; those that did not, became extinct. Needless to say, sleep researchers are far from knowing which such vital advantage is.

## 2. Sleep: Behavioral and Electrophysiological

In behavioral terms, sleep is associated with behavioral quiescence, raised sensory thresholds, easy reversibility, specific sleeping sites and body positions, it is a pleasing state and is dependent of circadian and homeostatic regulation [12,13,14,15,16].

Sleep may be also defined according to electrophysiological criteria. This allowed the recognition of two sleep states in mammals and birds, NREM and REM. However, the present report will maintain that mammals and birds followed separate evolutionary pathways. Nevertheless, we will only deal with the behavioral aspects of the mammalian sleep, aiming at elucidating how, when, and why the mammalian sleep appeared. After clarifying this question and its associated traits, we will end justifying our answer to the basic mystery: why we sleep?

## 3. Sleep: Monophyletic or Polyphyletic?

Our first task should consist in analyzing whether the evolution of sleep is monophyletic (it only appeared once) or polyphyletic (it appeared several times in different animal groups). As we already said, most authors referred in the first paragraph of this report believe that sleep is monophyletic, that is, NREM and REM would have evolved from a primitive state showing some kind of mixture between the two states. Oppositely, if it was polyphyletic, the two states would have appeared in two reduced samples of animals, as independent evolutionary adaptations. Therefore, the origin of sleep, and even the definition of sleep, would vary in function of the presence or absence of NREM and REM. The issue might be purely semantic: we may call true sleep the state that can be subdivided into two sub-states—the sleep of mammals and birds (adding the presence of the two states to the eight traits defined in the first paragraph of Section 2) and decide that those animals lacking the two states—poikilothermic vertebrates and invertebrates—show no true sleep. Nevertheless, after considering the huge neuroanatomical and functional differences, is not easy to consider a common origin for NREM and REM as well as for the sleep of animals showing a single sleep state. Therefore, the possibility of an independent origin may be worth of study.

## 4. The Origin of Mammals

Amniotes were the first vertebrates that, by using cleidoic eggs, were able to complete their entire life cycle on dry land. Contrasting with water, the properties of air—low specific heat, low thermal conductivity and abundance of oxygen—allowed amniotes high metabolic rates and thermal independence from the environment. Indeed, some descendants of the first amniotes, sauropsid and synapsid reptiles, attained high body temperatures (BT) by ectothermic procedures, i.e., heliothermy and tigmothermy [17]. Because of their advantages, terrestrial amniotes became dominant from the Triassic period onwards [18]. It should be noted, however, that the heliothermic thermoregulation of basal amniotes only was possible during daylight time. Therefore, those reptiles were, as well as modern basal reptiles still are, diurnal, and they must search safe refuges to rest in a thermo-conformist state during dark time. Therefore, the terrestrial life allowed amniotes some activity levels previously unseen, and quickly flourished in a variety of body sizes and types of life.

**Figure 1:**
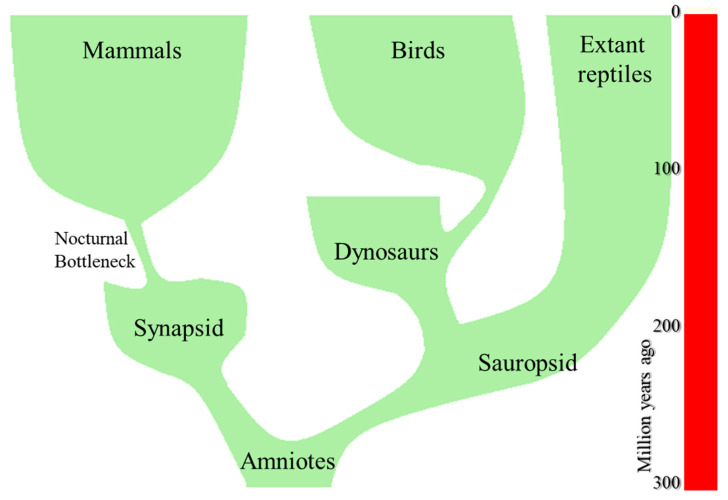
Phylogenetic three of amniotes. At present, three main branches exist: modern reptiles, birds-(including crocodiles) and modern mammals, that appeared after a nocturnal bottleneck.

Sauropsid amniotes were the ancestors of modern reptiles and birds, while synapsid reptiles, were the ancestors of mammals (Figure 1). The last ones constitute a monophyletic group that appeared around the Early Triassic, ~200 million years ago [19,20,21,22]. Like basal amniotes, pre-mammalian reptiles were ectotherms, strictly dependent on solar heat to warm their body. However, a few descendants of the primitive synapsid stock increased their energy metabolism. This provided them with a new source of metabolic heat and extended their activity, first to crepuscular hours, and then, with full endothermy, were capable of remaining active during the entire night. So, they filled a nocturnal niche that was empty of small sized vertebrates.

## 5. How Early Mammals Reached Endothermy

Modern reptiles possess the basic cardiovascular metabolic mechanisms and anatomic structures for endothermic generation of heat [23]. They may use muscular activity to increase the production of endogenous heat [24,25]. Some reptiles possess subcutaneous lipid layers providing thermal insulation [26,27]. This was furtherly improved by developing, hairy epithelial teguments [28] as well as dermal glands that evolved first, as sweating glands to avoid hyperthermia and, in specific anatomical places, became modified for milk secretion [29]. Likewise, some modern reptiles possess heat saving countercurrent vasculature in the limbs [25]. Therefore, it has been affirmed that the thermoregulatory difference between reptiles and mammals is more quantitative than qualitative [30]. Indeed, some extant turtles can maintain a relatively high BT even when submerged in cool water [26,27]. Likewise, tegu lizards may increase their metabolism to maintain a high BT through the reproductive season, so extending their activity to crepuscular and dark time [23]. Of course, attaining homeothermy was the result of trading off the advantages of filling a void ecological niche, for the metabolic costs of endothermy. Indeed, the metabolic efficiency of ectothermy is exceedingly higher than that of homeothermy [31].

### 5.1. The Control of BT in Reptiles and Mammals

**Figure 2:**
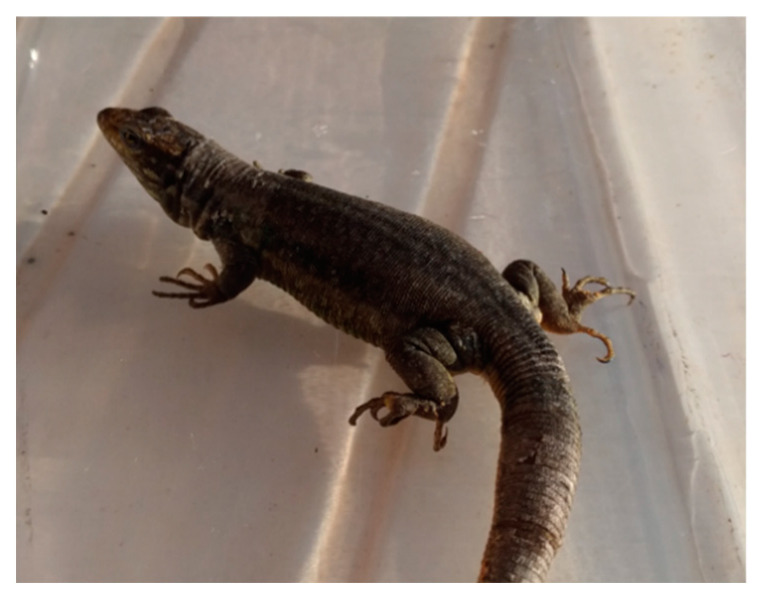
A 30 g alert *Gallotia galloti* lizard in RAB attitude after heliothermic warming.

**Figure 3:**
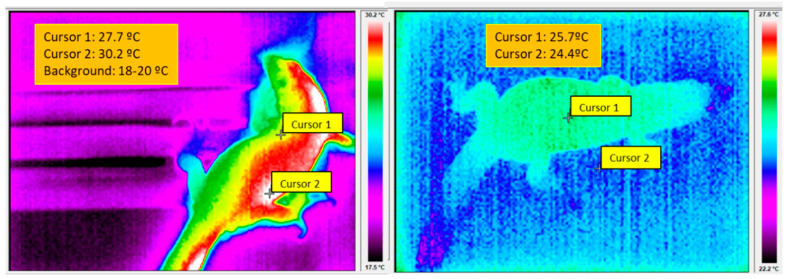
Thermographies of the same lizard of Figure 1. (**Left**): at noon, the heliothermic warming (BT = 30.2 °C, cursor 2) allowed full activity. (**Right**): thermography of the same lizard taken in total darkness, during midnight. The lizard had to be passively warmed during a few minutes before taking the right side image. Otherwise, its BT would be identical to that of the background and the animal would be completely invisible under the infrared camera. Nevertheless, despite the increase in BT (25.7 °C, cursor 1), the animal remained immobile in a cataplexic state, making no escape attempts. We attribute the immobility to circadian-related dormancy. Lateral color bars: **left**, from 30.2 to 17.5 °C. **Right**, from 27.5 to 22.2 °C. Thermographies taken by an Infrared Irisys IRI 4010 Camera.

Although extant reptiles show a wide variety of type of life and physiological adaptions, the BT control of basal reptiles depends, during light time, on two set points. A low set point determines entering in thermoregulatory heliothermic Basking Behavior (BB) to reach the BT needed for optimal physiological performance. However, on arrival to a second, higher set point, BB is substituted by Goal Directed Behavior (GDB) through an alert intermediate phase of Risk Assessment Behavior (RAB) (Figure 2 and Figure 3 left). After recognizing the safety of the immediate environment, reptiles begin the GDB behavior that allows performing vital activities: foraging, finding reproductive partners, escaping from predators, etc.

Noteworthy, the reptilian control of BT is placed in hypothalamic regions [32,33,34,35,36,37]. On the other hand, the BT is also dependent on environmental, internal, and constitutional factors. Among the first ones, the air temperature, rainfall, solar radiation, humidity, season, time of day, and predatory danger are important. Regarding internal factors, the thermoregulation also depends on gender, reproductive state, energy reserves and, most important, on body size [38,39,40,41] However, when the sum of environmental, internal, and constitutional variables become inappropriate for the optimal physiological performance—for instance, during night time—reptiles abandon the control of BT to enter in a thermo-conformist resting state that may persist for extended periods of time without harmful consequences [23,38] (Figure 3 right).

In mammals, the control of BT also depends on hypothalamic structures [42,43,44,45]. As in reptiles, the mammalian control of BT also depends on the temperature recorded by cool and warm sensitive thermoreceptors present in the skin. However, mammals attained a much higher control of BT, with an almost complete independence from environmental temperature [46]. We see, however, an evident continuity between the thermoregulatory control of reptiles and mammals.

### 5.2. Do Reptiles Sleep?

Many authors currently call sleep to the nocturnal rest of reptiles. Indeed, reptiles seem to show, during nighttime, most of signs of behavioral sleep. However, most experimental studies affirming that reptiles do sleep used inappropriate housing methods.

A researcher attempting to study the neurophysiological and behavioral traits of the eventual reptilian sleep, must give particular attention to the daily and seasonal thermal cycles [47,48,49]. For instance, it has been repeatedly observed that, in their natural environment, typical reptiles select cool burrows during dark time, where they remain hidden in a torpid state of “voluntary hypothermia”, with poor motor coordination and relative helplessness (Figure 3 Right) [49,50,51,52,53,54,55] Obviously, the behavioral rest, the raised sensory thresholds, the preference for certain body positions and “sleeping” sites can be easily explained as passive consequences of the cool-related torpidity of nighttime. This has been ignored in many reports in which reptiles are kept at constant temperatures during day and night, unknowing that, keeping reptiles during dark time at the *preferred environmental temperature of light time*, may compromise their health [56,57,58,59] that may arrive up to death [60,61]. Therefore, those studies that disregard a correct thermal housing may lead to faulty results [62].

Indeed, NREM and REM during nighttime has been claimed in bearded dragons [63] and, with less clear results, in tegu lizards [64]. Nevertheless, a more recent report [65] doubted the presence of REM in reptiles. Nevertheless, both Shein-Idelson et al [63] and Libourel et al., [65], persisted in affirming that sleep exists in reptiles. However, one of the authors never mentioned the above mentioned report of [37], while the second misquoted it affirming that “*sleep has never been recorded*” (in Tegu lizards) “*with the exception of three studies focused on circadian rhythms*”. However, one of the supposed study “focused on circadian rhythms” was the report of [37] that analyzed sleep, dormancy, quiet wake (quiescence) and active wake (with motility) in Tegu Lizards exposed to three environmental temperatures (17 °C, 27 °C and 37 °C). They found that, in electrophysiological terms, the state that is currently called sleep in reptiles was undistinguishable from dormancy, i.e., a state showing low BT, reduced activity and increased sensory thresholds, with no difference across EEG frequency ranges. They only observed a gradual decline in EEG amplitude, proportional to the activity levels and BT, agreeing with early authors [66,67,68]. In conclusion, [37] recognized that the signs of the currently presumed sleep-wake transitions were only progressive increases or decreases in electrographic, metabolic, motor and cardio-respiratory variables which always were correlated with BT, as expected from the Q_10_ law. This law explains that, with a conservative Q_10_ = 2 the physiological constants of a lizard kept at 10 °C are a 12.5% of the same constants at 30 °C. Furthermore, [37] only observed sleep (remember: undistinguishable from dormancy) in a few lizards exposed to the lowest tested BT (17 °C). A second report, also ignored by [63] as well as by [64] is that of [69]. These authors also studied tegu lizards but only found seasonal and daily correlations between BT and activity. In other words, tegu lizards, and, presumably, all basal reptiles, only show gradual changes that are dependent on circadian and seasonal oscillations in environmental variables and BT. We should remember here that the bearded dragons of Shein-Idelson et al. [63], were kept at a *constant* environmental temperature of 28 °C, while Libourel et al. [64] kept their animals to *constant* 27 °C. Contrasting, Piercy et al. [37] never recorded “sleep”/dormancy at BT surpassing 17 °C.

These results mean that the so-called reptilian sleep is not true sleep. It is only a passive consequence of the lethargic hypothermia obtained from recordings performed in animals maintained under wrong housing conditions. Similar lethargic effects, including reductions in EEG amplitude, are currently observed in hypothermic humans [70,71,72,73] but in no case have been equated to sleep, neither in neurophysiological nor behavioral terms. It is well-known that the mammalian sleep is a consequence of high activity in specific brain regions [74,75], quite different from the reptilian *passive*, BT-related, dormancy that involves the entire body, including brain (Figure 3 right, but also [37,69]. To conclude, no firm evidence supports the existence of true sleep with rapid reversibility in reptiles i.e., with one of the most important traits of sleep defined in Section 2.

## 6. The Nocturnal Bottleneck

It is believed that most specific behavioral and physiological traits of mammals evolved as a consequence of the so called “Nocturnal Evolutionary Bottleneck” (NEB). It proposes that some small sized reptiles shifted their activity from day to night, to avoid predation from co-existing diurnal bigger sauropsids. So, those pre-mammals exploited the nocturnal ecological niche that, by then, was empty of small sized vertebrate animals [76]

An evolutionary bottleneck is defined as an event that drastically reduces the size of a population (Figure 1) and only allows the survival of a few individuals carrying a small number of specific genes [77]. In the case of the NEB, Walls observed that, irrespective of their current chronotype, modern mammals show numerous adaptations to nocturnal vision. Accordingly, Walls concluded that mammals evolved through a prolonged (over 100 Mya) nocturnal phase that still holds in many modern mammalian species. Several authors provided additional supporting evidence for the NEB [78,79,80,81,82,83,84,85,86] and, at present it is considered a practically undisputed fact.

### 6.1. Adapting the Reptilian Vision to Nighttime

The mammalian ancestors were sight-oriented reptiles which, like modern ones, possess well-developed eyes, sophisticated tetrachromatic color vision, fine visual acuity and protection against ocular photo oxidation [87,88,89,90]. As the chromatic contents of the scarce amounts of nocturnal light are low, chromatic discrimination also was reduced in pre-mammals. So, their visual system became adapted to the scotopic environment [91]. As an example, nocturnal fruit bats only show a 0.4–0.6% of cones, while diurnal squirrels, well adapted to photopic vision and chromatic discrimination, possess ~86% of cones in their retina [92,93]. Moreover, early mammals were—and many modern ones still are, dichromatic—and even monochromatic—at variance from the tetrachromatic vision of their reptilian ancestors, [91,94]. Most important was that, pursuing the maximal visual sensitivity—needed for nocturnal vision—pre-mammals abandoned the filters protecting their eyes and retina from photo oxidation [95,96,97]. However, these changes determined high risks for the casual exposure to diurnal bright light [80,82]. Indeed, the light of high intensity in the wavelengths of maximal absorption of retinal pigments, is harmful to unprotected optical structures and retinal cells [98,99,100,101,102]. For example, despite being rather well protected by filters against light in the short wave range, actinic keratosis, cataracts, and macular degenerations are common conditions in aged humans [91,95,96,103,104]. Likewise, irreversible retinal damage has been found in rats exposed to quite low intensities of light: 60 Lux, in pigmented rats, possibly adapted to mesopic vision, and 20–25 Lux, in albino rats, better adapted to darkness [101,102].

### 6.2. Bottleneck: Nocturnal or Crepuscular?

Although the NEB hypothesis has been recognized by many authors, mice and other nocturnal mammals possess visual opsins sensitive to short wavelength light that, probably, allow some degree of chromatic discrimination. This fact promoted the proposal of an alternative “crepuscular bottleneck” hypothesis, presumably adapting the vision of early mammals to mesopic environments [85,86,105,106,107,108]. However, the existence of color sensitive pigments is insufficient to negate a nocturnal way of life. Indeed, few, if any, nocturnal animals show total absence of cones, nor their corresponding photopsins. This is the case of the nocturnal fructivore bats. Likewise, the aye-aye, a strict nocturnal species, retains the gene for a short-wavelength opsin which should be indicative of dichromatic color vision [92,109,110]. To summarize, a high proportion of rods is necessary, but not sufficient, to exclude the existence of photopic vision. Conversely, even retaining a significant proportion of cones is compatible with a nocturnal (or crepuscular) mode of life [92].

We used the adjectives “nocturnal” or “crepuscular” for labeling the bottlenecks. However, such labels also apply to the properties of the visual systems, which certainly can be adapted to diurnal, nocturnal, or crepuscular modes of life. Furthermore, these adjectives also express quantitative variations. For example, a retina may be adapted to pure nocturnal (scotopic) vision by showing only rods, all with a single rhodopsin pigment. Contrasting a pure diurnal (photopic) retina only would show cones, with a variety of photopsin pigments that would allow chromatic discrimination. It should be noted, however, that a huge majority of retinas—if not all—can work in mesopic conditions, i.e., in intermediate levels of the scotopic-photopic dimension, using rods and cones accordingly. This said, the original NEB hypothesis [76] assumed that primitive mammals were adapted to exclusive nocturnal life, affirming that the reptilian retinas, that began being photopic, attained, along progressive adaptations to reduced amounts of light, an almost pure scotopic vision [85].

Under these premises, we may analyze the conceptual differences between nocturnal and crepuscular bottlenecks that some authors consider incompatible with each other. The crepuscular vision could in fact be more efficient for performing some activities under low intensity of light, for instance, feeding or predation avoidance [111]. However, considering the purpose of the present review—understanding how the mammalian sleep was originated—we will see that the difference between the two bottlenecks is irrelevant. In both cases, the process involves costs and benefits. [112,113,114]. However, while the benefits would be different in crepuscular and nocturnal environments, the costs (blindness) would only be important during daytime and would be identical for both types. In other words, the filters were abandoned to improve the adaptation to dark, but in both crepuscular and nocturnal bottlenecks, the adaptation to the dangerous bright light was, in practice, identical: zero. Therefore, early mammals had to remain resting in lightproof burrows during light time, irrespective of their bottleneck type.

### 6.3. Adapting Non-Visual Sensory Systems to Nighttime

Early nocturnal mammals compensated for the reductions in visual performance by developing alternatives for orientation in the dark. The auditory sensitivity was greatly increased after modifying the reptilian articulation of the jaw and relocating the residual bones in the middle ear, where they became the malleus, incus, and stapes. These changes also extended audition to high frequency ranges [115,116,117]. The development of the ear pinnae, with its motor control, allowed locating the precise origin of sounds and the capacity to follow the movement of the sound sources [118,119] Furthermore, the development of mystacial vibrissae and the associated sensory receptors allowed the so-called “face touch”, facilitating exploration in the dark [120].

However, the most important consequence of the NEB was the improvement in the sensitivity and analytical capacity of the olfactory system. The skull endocasts of successive early mammals show continuous size increase in olfactory bulbs [117,121] correlated with increased telencephalic and cerebellar size [122,123]. The improvement of olfaction imposed high demands in anatomic and computational resources for creating and memorizing precise navigational maps, to find foods, recognizing dangers, friends and relatives, and is currently considered capital for the development of the distinctive mammalian isocortex [124,125]. As Rowe [117] summarized, “*the ancestral mammal was a tiny terrestrial creature that scurried and climbed over complex three-dimensional surfaces of its microhabitat, carrying its young in a pouch, and nurturing them with milk and warmth until they were self-sufficient in feeding and could regulate their own body temperatures. With a* (well-developed) *neocortex and corticospinal tract, it was exceedingly agile and quick and used olfaction in navigation, scent-tracking, and myriad social behaviors*”.

## 7. The Birth of Mammalian Sleep

The previous paragraphs explained that, to minimize the risk of extinction, early mammals remained paralyzed with closed eyes in lightproof burrows during daytime for ~100 million years. This was how the mammalian sleep was born. This is, probably, the most important result of this report.

### The Origin of the Sleep Regulation

The homeostatic and circadian regulatory processes are almost unnecessary in reptiles. First, reptiles maintained under constant darkness show free running circadian cycles in body temperature and activity [126]. However, the daily cycles of light and dark cycles possess sufficient intrinsic power to force the day-night switching between rest and activity as a simple reactive response, or, at least, non-necessarily proactive. Obviously, no reptile would be capable of remaining active on the arrival of cool nighttime, irrespective of having or not a functional internal clock. Furthermore, if several consecutive cool and cloudy days appear, reptiles have no problem in suppressing their activity for several days, and even weeks without harmful consequences, so remaining inactive inside their burrows [23,38]. Therefore, they must ignore, during such intervals, the eventual presence of circadian clocks and the homeostatic drive for regulating the amount of resting time. Indeed, the impending dusk and coolness are, by themselves, sufficient to impose rest during nighttime and during cloudy-cool days. We should thus conclude that, in reptiles, the eventual homeostatic systems regulating the cycles of rest and activity are rather weak and can be ignored—are in fact ignored—in many cases. On the contrary, the mammalian homeothermy imposed the substitution of the weak reptilian thermal Zeitgeber [127], by a stronger, immutable Zeitgeber, the environmental light-dark oscillations [113,128,129]. Otherwise, given the constancy of their BT, the maintenance of the old thermal reptilian Zeitgeber, would have permitted activity around the clock, with undesirable consequences.

We already described how the nocturnal pre-mammals escaped from the dangers of bright light by developing an active immobility and adopting stereotyped resting positions inside well-protected dark burrows. Those pre-mammalian reptiles also raised their sensory thresholds during resting time, to avoid reacting to trivial stimuli. Vision would serve for nothing in animals that rested in dark burrows. Furthermore, the visual shutoff would save significant energetic and computational resources. In humans, over 50% of the cortex is directly or indirectly related to visual analysis [130,131,132,133]. The costs of maintaining vision during dark time may be so high, and the advantages so small, that probably explain the universal sleep-related eye closure. So, developing a strong circadian regulatory system was necessary for guaranteeing immobility before perceiving the first lights of dawn. The blindness risk was furtherly reduced by developing a hypothalamic flip-flop switch [134] that would guarantee the stability of the sleep/activity cycles.

Contrasting with vision, maintaining audition was highly adaptive in dark burrows, in which the sound may travel freely. Therefore, a full acoustic sensitivity during the new rest-sleep state was adaptive in early mammals and continues being active in sleeping modern ones [135,136,137]. To end, cooled reptiles are still capable of weak responses to strong stimuli. Instead, mammals, with a constant BT, became capable of rapid and efficient motor responses, i.e., a rapid state reversibility.

Summarizing, early reptiles, but also modern ones, may have circadian and homeostatic controls of low efficiency that can be easily circumvented because of environmental factors. Instead, mammals developed efficient circadian and homeostatic mechanisms that made impossible the escape from their activity-rest circadian cycles. In this way, they invented sleep, a behavior that depends on circadian and homeostatic controls that, in early mammals, forced sleeping during daytime and wakefulness during nighttime. This chronotype, however, was inverted when some mammals returned to the diurnal niche to fill niche that remained void after the dinosaurs’ extinction.

## 8. The Variability of Sleep and the Evolutionary Pressure

High levels of mammalian diversification began in the Cretaceous. The early insectivorous diet was substituted, by scavenging as well as by predator and carnivorous lifestyles [82,138,139,140,141,142,143,144]. Possibly, however, these early diversification events were dead-end evolutionary experiments, far from the mainstream of diversification that took place in the Cenozoic [145] because of the K-Pg extinction event [146,147,148,149,150,151,152,153]. Such extinction left free the diurnal niche and allowed Cenozoic mammals to develop a wide variation in chronotypes, alimentary modes and body sizes.

It is important to remark on here the immense variability that many authors recognize in the current sleeping modes [154,155,156,157,158]. The small size and the diurnal rest were certainly vital for the first mammals during millions of years. However, recovering the ocular protective filters was compulsory to conquer the diurnal niche. In this way, mammals ended with widely different modes of sleeping—diurnal, crepuscular, nocturnal, and even cathemeral. However, they maintained the essence, that is, the behavioral quiescence during a part of the daily cycle. Therefore, the evolutionary advantages of every one of the newly developed traits of sleep variants was—still is—minor. Indeed, their high phenotypic variability may be a paradigmatic example of an old, well-known, and intuitive principle of evolutionary biology: “*the genetic and phenotypic variability is inversely related to the intensity of stabilizing selection*” [159], a principle that has been incorporated to texts of evolutionary biology [160]. As an easy example, the color variability of modern cars is infinite, but no car exists without wheels. Therefore, the relative importance of wheels vs. color is undisputable. Applying this to behavioral sleep, we know that it shows eight basic traits, but the single one that is truly discriminative and never fails to show, is the behavioral quiescence. Indeed, the other seven traits show multiple variations in different species. Their role for adaptive performance may be, therefore, as irrelevant as the color of cars.

## 9. Comparing Behavioral Sleep and Rest

Still analyzing the relative importance of the eight traits used to define behavioral sleep, we observe that six of them—quiescence, reversibility, body positions, resting places, and circadian organization (Section 2) can be found, not only during sleep, but also in resting wakeful animals. Continuous oscillations in sensory thresholds can be found in function of the levels of reticular activation [145,161,162,163,164,165,166]. Contrasting, the homeostatic rebounds observed after sleep deprivation have been considered exclusive and unequivocal signs of sleep. The following paragraphs will analyze the importance of this trait.

### 9.1. Wakeful Rest and Laziness

Motor rest may appear as passive adaptations to environmental factors. For instance, poikilotherms, but also hibernating animals, show cool-related inactive periods. Animals also rest to recover from fatigue, after heavy meals, or after intense muscular exercise. They also remain immobile when stalking their next meal or freeze after sighting a predator.

In many cases, however, the reasons why animals rest, cannot be discerned. Indeed, zoologists often are puzzled after observing that the time devoted to foraging, reproductive, or defensive activities only occupies a small part of the day, and a part of the daily time remains as idling without recognizable function. After observing the abundance of resting periods whose utility cannot be easily explained, they were dubbed as “lazy” [167] However, the word “lazy” has heavy anthropomorphic and moral loads. Therefore, it has been substituted by “idleness”. Interestingly, it can be said that idling animals may neglect foraging, procreation, and may even show increased predatory risks. Therefore, it seems that idling is non-adaptive and should have been disposed of by natural selection. However, the abundance of idleness in animals and humans is so puzzling that the mystery of idling parallels the mystery of why all animals do sleep.

### 9.2. The Principle of Stringency

The abundance and adaptive value of idleness has been satisfactorily explained after the “Principle of Stringency” (PS): “*Time-energy budgets evolve to fit to the times of greatest stringency*” [168]. Although the total time needed for vital activities might be less than 24 h in epochs of surplus, animals do not try to maximize their biological efficiency by using the excess of available time to increase food intake and reproductive efforts. The genotypes committed to excessively rapid body growth and reproduction would enjoy a temporary advantage during periods of abundance, but would suffer severe setbacks during hard times, possibly leading to extinction. Therefore, maximizing the immediate advantages of surplus epochs is a short-sighted strategy, and animals evolved to keep a prudent use of their time-energy budget during favorable epochs, accommodating it to the predictable demands of shortages [168]. Hence, they spend the excess time [167]. This explains why wakeful predators often ignore killable preys, why foragers often ignore food, and why species engaged in ceaseless reproductive activity do not exist. At present, the importance of the PS is well recognized among zoologists and ecologists [167,169,170,171].

### 9.3. The Idling Wakeful Rest

Increased interest has been observed, in recent years, for the state of Waking Rest [166,172]. This state is, in fact, equivalent to the idling rest described in the previous paragraph. In the following, we will join them under the name of Wakeful Idling (WI).

Not surprisingly, the proportion of time devoted to unproductive (lazy) activities has been carefully ruled since immemorial times and in all human cultures. WI has been observed, not only in mammals, but in many other animal groups, and even in insects [173,174,175,176]. The time devoted by professional workers to uninterrupted tasks, for example driving, is carefully regulated by traffic rules. Indeed, trucks and coaches must be compulsorily equipped with chronographs that measure the total driving time and mark the need of periodic recesses, a feature that, in modern tourism cars is also used to warn on the convenience of taking driving pauses. The need of pauses is also important in school, in which, the productive work of kids must be compulsorily interrupted by periodic recesses. Even more, it has been found that the tendency for lazy WI is innate [177,178,179,180] and the periodic oration time is even a religious command.

Currently, the periods of WI occur in a time scale of minutes. However, they can be expanded, to days and even weeks, in holidays. We spend huge amounts of time, money, and resources on unfruitful activities that may be qualified as lazy-idle. Such qualification, however, does not mean neither absence of function, nor objectionable behavior. Instead, the bulk of evidence points to the adaptiveness of WI and the convenience of distinguishing it from sleep and active wake [181].

Interestingly, WI shows a highly significant hedonic dimension: humans and animals with capacity for hedonic experiences, enjoy WI and, undoubtedly, these activities are only acceptable while they are pleasing. However, following the lead of [182,183,184], we know that pleasing stimuli are those that facilitate the homeostatic balance, the survival and the propagation of the species. Reciprocally, displeasing stimuli are always related to homeostatic imbalance and impaired survival. So, the pleasure of doing WI must be indicative of positive adaptation, increasing the survival chances and the biological efficiency. We must conclude—and this conclusion is extremely important—that WI, together with leisure activities, are but rebounds of rest that compulsorily—by legal rules, but, most important, by physiological and psychological constraints—are interspersed between periods of productive wakefulness. Therefore, we must recognize that the full set of seemingly unprofitable laziness is homeostatically regulated, with well-defined rebounds after prolonged periods of productive activity. This is another important finding of this report. It has been obtained, not from experimental procedures, but by no less significant historical and cultural evidence. Indeed, it is impossible to imagine a world completely devoid of WI.

### 9.4. Pleasure, WI, Sleep, and Homeostatic Regulation

The relationships between pleasure and homeostatic regulation have also been extended to sleep: if sleeping is pleasing for humans—sleep has been called “*the gentle tyrant*”—and, if the unpleasantness of sleep deprivation is the basic factor determining the daily unstoppable sleep propensity, pleasure must be the link responsible for sleep homeostasis [16]. By the same token, if WI is pleasing—and certainly, it is, for humans, dogs and cats and many other animals—the pleasure of resting lazily must be the link controlling the homeostatic regulation of WI. Even more, a growing number of studies demonstrate the equivalence between WI and sleep for facilitating the consolidation of memories [185,186,187,188,189,190,191,192,193,194,195,196]. Therefore, it seems that WI is important for many essential activities.

We arrived at a point in which the set of traits currently used to define behavioral sleep and rest are in fact hardly distinguishable. Both show quiescence, raised sensory thresholds, reversibility, preferred resting/sleeping locations, similar relaxed body positions, circadian organization, pleasure and, as we observed, both are dependent on homeostatic regulation. Moreover, the WI is the first and imperative sign of impending sleep [197,198]. So, sleep and WI are inextricably tied, up to the point that we would dare to affirm that sleep is an upgraded version of idling.

Of course, important differences continue existing between WI and sleep. An example is the consciousness-unconsciousness dimension. While consciousness is maintained during WI, it is deeply altered during sleep. We also mentioned the eye closure that is rare during WI and compulsory during sleep. Nevertheless, many people, but also many institutions, join sleep and idling, when asserting that excessive sleep is a sin, indicative of lazy personality. Furthermore, we should remember that sleep was invented by early mammals to guarantee immobility during light time. Thus, it can be said that the primary function of sleep was guaranteeing immobility during a part of the daily time and, there is no reason to negate that, at present, may continue being so. Such a proposal is not new [199,200,201,202,203,204,205].

We must recall, however, the most important difference between WI and sleep. While sleep must be necessarily interspersed between wakefulness periods, we know that, for example, retired people may show indefinite time idling, that is, we know no limit for the time spent in WI. We should ask, therefore, why the homeostatic regulation of WI allows unlimited WI time, while the maximal duration of sleep in healthy individuals is always limited. Obviously, this question is equivalent to ask how the homeostatic regulation of sleep works. The following lines will try to defend that the homeostatic regulation of sleep may be disputable. In fact, it has been already challenged [206].

First, the compulsory need of sleep rebounds may determine the production of sleep during inappropriate circumstances, for example, interfering with the circadian regulation [207] Second, the cellular mechanisms of sleep homeostasis remain undefined [206,207,208,209]. Third, the nature of the regulated variable controlling sleep duration remains also unknown. Fourth, no relation has been found between the electrographic traits of the rebounds and those of long-term deprivation [210]. Fifth, no correlation has been found between sleep loss and the size of the rebounds. For example, Randy Gardner remained awake for 11 days (264 h), but it was fully recovered after 14 h 40 min, 10 h 30 min and 9 h on the successively following three days, [211]. To explain the lack of correlation between deprivation and rebounds it is currently affirmed that the slow wave EEG power, the duration and continuity of the rebounds are indicative of increased sleep “intensity”. However, this is, most likely, an “ad hoc” argument, almost impossible of refutation. Furthermore, it is hard to explain how Randy Gardner, may have had an “ultra” intense sleep, enclosing, within ~33 h—the total recovery time—the 264 h of lost sleep.

Even the impossibility of total sleep deprivation for long periods without rebounds of time has been challenged. Indeed, the universality of the lethal consequences of sleep deprivation is, either weak or absent [212,213]. Given that inescapable stressing stimulation is, in practice, the single procedure to provoke sleep deprivation, the development of Learned Helplessness that currently appears after inescapable punishments is a syndrome well-known among psychologists [214] and may explain the lethality of total sleep deprivation in rats [202]. So, one might say that the cause of death in insomniac rats was not sleep, but the development of the Learned Helplessness syndrome. Furthermore, extended periods of total absence of sleep without rebounds and without observable harmful consequences, have been found after parturition in marine mammals and their calves [215], in the Ganges dolphin, that never stops swimming [216], and, probably, in migrating mammals (elephants: [217]); herbivores: [213]).

In summary, the homeostatic regulation of sleep demands the existence of an unknown neural system that registers the amount of sleep lost, modifying then the levels of an unknown variable that, on arrival to a certain unknown threshold, promotes the activity of an unknown feedback mechanism organizing the size of the rebound. As a side result, of unknown cause, such mysterious mechanism increases the delta EEG power, determines the negative mood and the impaired psychological performance of the sleep deprived subjects and, after the rebound, returns the unknown variable to its previous levels. So, we would qualify the complete set of mechanisms involved in the homeostatic regulation of sleep, as the result of a complex set of mysterious mechanisms that have evaded, over 40 years, the attempts of identification.

So, an excessive number of problems exists involved in the maintenance of the up to now inviolable belief in the homeostatic regulation of sleep. But, if anybody would dare to negate it, he should search sound alternative explanations for the rebounds, as affirmed by Siegel [204] and Frank [206].

An interesting idea was proposed in an early preprint version of [218] However, the word “punishment” (see next paragraph) was retired from the final version [219]:

*“Is sleep rebound a way to make up for a loss of an otherwise impaired biological process, or is it instead merely a “punishment” phenomenon, evolved to guarantee that a constant, largely species-specific amount of sleep is met?”*.

[218]

The idea of punishment was also, but independently, proposed by our group [16] to express the highly negative hedonic value of sleep deprivation, contraposed to the positive value that most humans assign to sleep. Indeed, the sleep deprivation is one of the most excruciating tortures and, conversely, most people consider sleep as a highly pleasing state. It should be noted, therefore, that the rebound, as expressed in the words of Geissmann and collaborators is never a “punishment”. It is, instead, the pleasing recovery of the lost sleep provoked by stressful (punishing) stimulation. So, we would rephrase the sentence of Geissmann and collaborators by affirming “that the sleep loss and the insomnia may constitute a punishment evolved to guarantee that a species specific amount of sleep is met”. And we would append that “the rebounds, when possible, are the reward that evolved to reinforce the recovery of sleep loss”. This is, in fact, what most people do when joyously extend their sleeping time in Sundays, spending their free time in joyous idling.

It should be noted, however, that the deprivation and the rebound are not “merely” rewards or punishments. We should ask immediately, why the sleep-related rewards and punishments exist. Since the already cited reports of Cabanac [182,183,184], we know that both rewards and punishments are responses to stimuli with high survival significance. Therefore, the inclusion of rewards and punishments in the regulation of sleep does not mean rejecting the homeostatic regulation of sleep. They are, instead, the missing and unknown mechanisms previously listed.

The neurophysiology of the brain rewarding and punishing systems is well-known since the seminal work of Olds and Milner [220] and the implication of reward and punishment in sleep has been extensively described [16] Therefore, if we join (1) the literature dealing with the physiology and the psychology of pleasure and punishment, with (2) the huge amounts of information on sleep physiology, we may obtain an easy and parsimonious explanation of the sleep rebounds. Simply, sleep is a pleasing state and sleep deprivation is displeasing. Dopamine is the chief neurotransmitter responsible of promoting wakefulness [221,222,223,224] and of the “wanting” of pleasing stimuli [225,226,227,228]. As a consequence, when the levels of extracellular dopamine descend, the drive for wakefulness also drops [229,230,231] and the wanting for sleep (the sleep propensity) raise. Therefore, under the impending descents in mesolimbic dopamine, animals fall asleep. Then, after a sufficient sleeping time, the levels of dopamine are restored, promoting so, the awakening of the subject [232,233]. Of course, in the case of sleep deprivation, the dopamine levels should suffer higher reductions, and the restoring time must increase [234,235,236], i.e., the rebound.

Of course, the complexity of the relationships between wakefulness, sleep, dopamine levels, pleasure and punishment cannot be explained with a short summary, as described in the previous lines. However, it is evident that the pleasing-displeasing explanation of the sleep deprivation-related rebounds is quite congruent with well-known facts and more parsimonious than the current hypothesis of an independent and specific homeostat for sleep.

Nevertheless, one may continue questioning why sleep is a pleasing state. An interesting answer might come from the necessity of the WI. Indeed, we may idle while we are awake, but, undoubtedly, we also idle when we are asleep. We already asked on the possibility of a world with no idling at all and we arrived to a rotund negative. But, if it is currently affirmed that sleep is universal in animals, we should add that idling is also universal. At present, humans pursue continuous activity in industries, commerce, communications and even in the leisure industry. We know, however, that the attempts for attaining such objectives fail in the weakest link of the chain: the human subjects. They show plenty of ailments because of the shift work. One would ask how we may alleviate these ailments: the answer is simple: increasing the amount of free time. At present there are many proposals of a down reduction in the working week to only four days, leaving, therefore, a long weekend that, most likely will be used in WI. Apart from reducing the unemployment, the health and happiness of people would be undoubtedly improved.

## 10. Summary and Conclusions

The present report highlighted several important questions related to mammalian sleep. First, we described a coherent evolutionary relate explaining the evolutionary origin of sleep, but only in mammals. Second, and far from what is currently believed, sleep resulted to be polyphyletic. Indeed, it must have appeared on several occasions in different animal groups, possibly, by evolutionary convergence. Small, pre-mammalian reptiles invaded the nocturnal niche that, by then, was free of small sized vertebrate life. They were forced to adapt their visual system to darkness for increased sensibility. This was achieved at the cost of abandoning the filters suppressing the most energetic fraction of the diurnal light and losing the protection against photo-oxidative damage. Therefore, early mammals were forced during million years to rest immobile with closed eyes, hidden in lightproof burrows during light time. This was how they invented sleep.

Noteworthy, the described processes constituted a second evolutionary bottleneck. The Walls’ bottleneck explains that a small group of pre-mammals became adapted to nocturnal life, and so they evolved until becoming full mammals. It also explains the persistence of visual adaptations to scotopic vision in the eyes of extant mammals. The adaptations were, however, insufficient; the survivors were submitted to another bottleneck: only a small number of individuals capable of inventing sleep during light time, survived; the remainder continued being nocturnal or became blind and extinct.

The adaptation to life in the dark forced the modification of the remaining sensory systems and favored the development of the telencephalic cortex. However, early mammals only attained complete freedom after the K-Pg mass extinction that freed the land from big sized competitors. Then, some mammals recovered the visual filters and were able to return to diurnal chronotype. This was the origin of the wide variability in chronotypes and sleeping styles.

The second part of this report has been devoted to the analysis of the behavioral properties of sleep. Perhaps, another highly significant contribution of the present report is the recognition that idleness—laziness—is not an objectionable behavior, but an adaptive behavioral strategy that is homeostatically regulated. Besides, we observed that the complete set of signs currently used to define sleep can also be used to define the idling rest during waking time, with only small—albeit important—differences. This adds evidence to the triviality of sleep, i.e., that it merely serves for guaranteeing rest during a part of the daily cycle as previously proposed by many authors [199,200,201,202,203,204,205].

The entire evolutionary relate exposed in this report can only be applied to the mammalian sleep. Indeed, no known nocturnal bottleneck has been described to explain, either the sleep of invertebrates, or that of poikilothermic vertebrates and not even the avian sleep. Therefore, the sleep of non-mammals must have appeared because of multiple processes of evolutionary convergence. In this case, however, science must analyze whether the diverse sleeping modes also serve for guaranteeing quiescence. If the need of quiescence would be universal, sleep would be also universal. But, if the function would be different in mammals and non-mammals, the universality of sleep should be denied, and the sleep-like state of non-mammals would be a state different from true sleep. Nevertheless, even in the case of being universal, sleep should be polyphyletic and should have evolved, at least, three times: two in mammals and birds, (but not in poikilothermic vertebrates) and, in a third wave—probably much earlier—in invertebrates. However, after considering the immense phylogenetic distance between different invertebrate orders [237,238,239], the number of independent sleep phyletic lines might be much higher.

## Data Availability

Not applicable.
